# Fourth-Order Quadratic Buck Converter Controller Design †

**DOI:** 10.3390/s24020557

**Published:** 2024-01-16

**Authors:** Gabriela-Madalina Pop, Ioana-Monica Pop-Calimanu, Dan Lascu

**Affiliations:** Applied Electronics Department, Faculty of Electronics, Telecmmunications and Information Technologies, Politehnica University Timisoara, 300223 Timisoara, Romania; ioana-m.pop@upt.ro (I.-M.P.-C.); dan.lascu@upt.ro (D.L.)

**Keywords:** controller design, coupled inductors, type III error amplifier, static conversion ratio, step-down converter

## Abstract

This paper aims to outline the process of dimensioning a controller tailored for a fourth-order step-down converter. In order to conduct a thorough small-signal analysis, it is imperative to find the state–space model in matrices form. Given its fourth-order nature, the control-to-output transfer function also aligns with this order, although its degree is ultimately reduced to a second-order using the tfest function. It is remarkable that the design of the type III error amplifier assumes a central position in the overall controller design process. The theoretical analysis was then subjected to rigorous validation via simulation, with particular attention paid to the step response in both input voltage and output resistance. This study developed from the desire to validate the efficacy of reducing the control-to-output transfer function degree using the tfest function, aiming to highlight a fourth-order converter to which controller design theory can be applied, related to that for a second-order converter.

## 1. Introduction

Buck converters, also known as step-down converters, are widely used in applications where a regulated voltage needs to be obtained from a higher voltage source. Examples of such applications are industrial applications [1,2], like motor drives or factory automation [3,4], telecommunications [5], solar power systems [6], automotive [7], and voltage regulators [8,9,10,11,12]. This ability to reduce the voltage level is essential in many electronic devices, and in the literature, different topologies, starting with the classical step-down buck converter [13], other types of non-isolated [14,15] converters or isolated step-down converters [16,17,18] can be found. In contrast to various other topologies resembling the buck configuration, the introduced converter from [19] showcases a notably smooth current profile at the input. Moreover, it is demonstrated to be particularly well-adapted for applications that need a minimal discrepancy between the input and output voltages. The efficiency of this innovative converter remains consistently excellent across a broad spectrum of duty cycle variations [19]. Single switch-based semi-quadratic buck converters are in the category of non-isolated converter types. This converter is able to provide a higher stepping-down conversion ratio than the classical one. It contains only one transistor, but the number of diodes is four, and the converter order is five.

The buck converter presented in [20] is a hybrid buck topology that exhibits lower losses at heavy loads and is used for charger applications in mobile electronics. Its disadvantage is that it has three MOSFET transistors. The authors from [21] are proposing a new type of switched inductor semi-quadratic buck converter that is composed of a semi-quadratic buck and a L-switching structure to obtain a higher voltage conversion ratio. This topology has the advantage that the conduction losses and the switching stress are lower compared to the two-switch semi-quadratic buck topology [22]. Another one-transistor three diode fourth-order quadratic buck converter is proposed in [23]. This topology exhibits good output voltage regulation and fast transient response, but the highest efficiency is only 82%. A maximum of 93.5% efficiency was achieved by the authors from [24] with a buck converter that has a cell made of two switches placed in parallel with two crossly connected identic capacitors and two inductors that are coupled. The interesting aspect is that the conversion ratio does not depend on the turn ratio of the coupled inductors. The topology of a high step-down bidirectional converter that also contains coupled inductors and two energy-transferring capacitors is described in [25]. This time, the turn ratio of the coupled inductors appears in the formula of the conversion ratio. A traditional quadratic buck converter (QBC) is presented in [26], and the traditional one-cycle controlled QBC and an improved version that is obtained by including the inductor current to diode voltage as an integral variable and introducing feedback of output voltage is reported in [27,28]. A series of quadratic step-down DC–DC converters is developed by invoking the principle of reduced redundant power processing. This involves a systematic approach that aims to improve the efficiency and performance of the converters through the reduction of unnecessary or duplicated power processing components. As indicated, the quadratic converters suggested in this context are formulated through the interconnection of fundamental switching converters in non-cascaded configurations. Although initially designed with two active switches, an analysis of practical implementations indicates that these converters can be adapted to configurations using only a single switch. The obtained converters thus represent an alternative approach to the conventional cascade solution [29]. In [30], a quadratic step-down converter is introduced. In contrast to various existing step-down solutions documented in the literature, this converter proves to be exceptionally well-suited for applications demanding an output voltage only marginally lower than the input voltage. The precise operation of the converter is thoroughly validated through a combination of simulations and experimental outcomes. Notably, the converter demonstrates an efficiency exceeding 90%. This is particularly noteworthy considering that, in comparison with the traditional buck converter, the proposed design incorporates an additional inductor, two additional diodes, and one extra capacitor. 

Compared to alternative quadratic topologies, the static characteristic of this particular design exhibits a higher step-down voltage difference between the input and output. Notably, this static conversion ratio is achieved while using only a single active switch, three diodes, and maintaining an equivalent number of inductors and capacitors [31]. The multiple-output synchronous buck topology is also part of the class of non-isolated buck converters. This converter achieves multiple independently regulated outputs with reduced switching components [32]. In [33], the primary focus is centered on the generalization from a two-stage to an *n*-stage stacked step-down converter. Emphasizing the significance of the DC conversion ratio, this study employs mathematical tools as a methodological approach to thoroughly investigate the fundamental properties associated with the converter. The multi-phase interleaved converter could also be a solution. For the multi-phase structure, the classical buck topology, synchronous buck [34], or in a particular case, for example, two-phase interleaved step-down with coupled inductors topologies can be used. In [35], the proposed topology can achieve a higher step-down ratio than the conventional buck by adding three coupled inductors and two switches to the interleaved two-phase buck converter. In the literature, the number of quadratic converters is very high. In [36], a switching regulator with a quadratic-based step-down topology used in hybrid electrical vehicles is presented.

In the topology presented in [37], isolation is achieved with the help of an optocoupler, increasing the frequency and current capability, and there is no need for the diode in the conventional circuit. 

In [38], the different converter topologies have been analyzed and compared in more detail than presented here in this short introduction. Therefore, it can be concluded that quadratic converters and coupled inductor converters are common types, and various topologies can be found in the literature.

The author of [39] proposed an ideal quadratic buck-type topology that is theoretically analyzed and verified by simulation and experiments. The present paper proposes a controller design for a fourth-order quadratic buck converter, which was obtained in [39]. This topology provides a higher conversion ratio at the same duty cycle when compared to the classical buck converter. To design this controller, the following steps were taken:

- Small-signal analysis was carried out using a state–space model;
- Linearization of the control-to-output function;
- Approximation using a second-order function;
- A type III error amplifier was designed;
- The feasibility of the type III controller was confirmed in the simulations.

## 2. Materials and Methods

**Figure 1:**
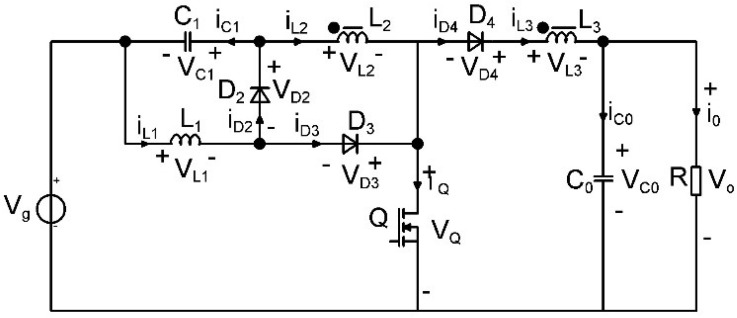
The boost−type topology converter with coupled inductors proposed in [40].

The process of transforming the boost topology proposed in [40] into a step-down converter is illustrated in Figure 1. It involves several sequential steps. To create the new converter, the initial step is to replace the existing semiconductors with single pole single throw (SPST) switches. Once this substitution is completed, the next step is to identify the switching cell within the circuit. In order to handle the switching cell effectively, the concept of cell rotation is employed. This technique involves rotating the extracted switching cell between the source, common, and load terminals. By doing so, the necessary changes are made to the circuit configuration to achieve the desired step-down functionality.

Upon obtaining the new converters at the SPST level through the rotation process, switch synthesis is invoked. During this phase, each SPST switch is replaced by a transistor or a diode, depending on the quadrants it is operating.

**Figure 2:**
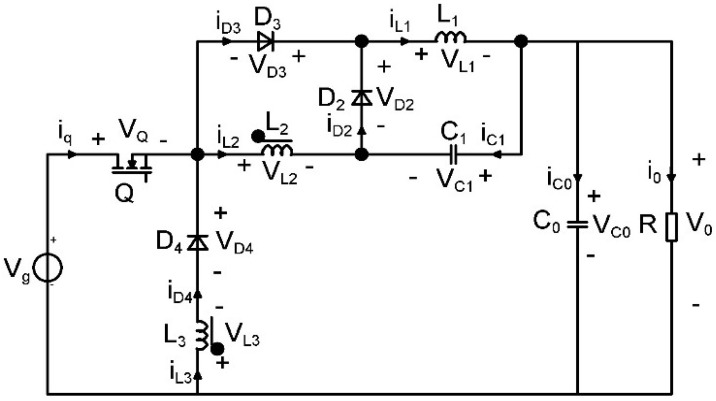
The buck−type topology with coupled inductors proposed in [39].

**Figure 3:**
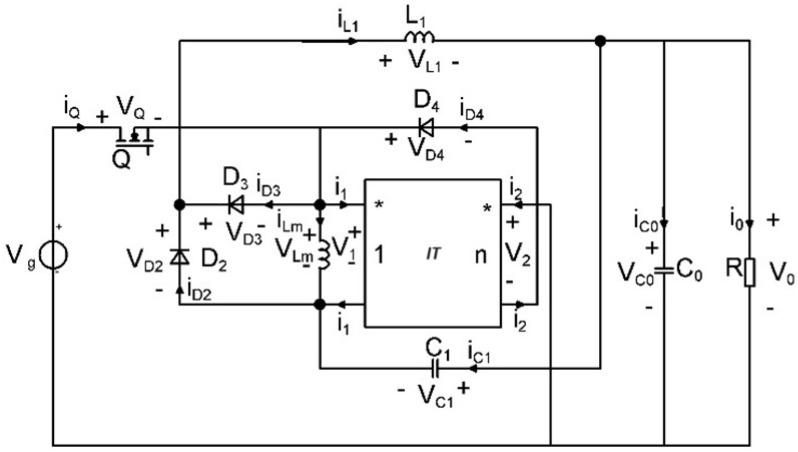
Equivalent model of the proposed buck−type topology with coupled inductors.

As a result of this process, two new single-transistor topologies are generated. The newly created step-down converter is presented in Figure 2 and its equivalent model is depicted in Figure 3.

The operation of the transistor is controlled by a pulse width modulation (PWM) signal, characterized by a duty cycle denoted by *D*. The switching frequency fs is constant, and the switching period is labeled as *Ts*. 

**Figure 4:**
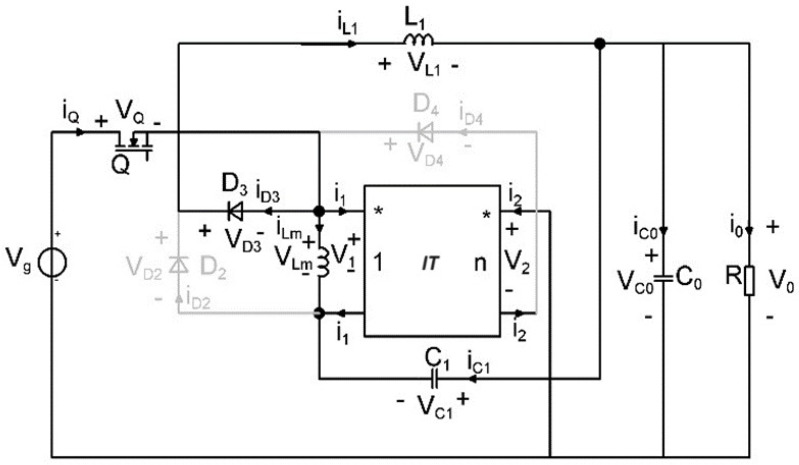
Equivalent model of the proposed buck−type converter with coupled inductors: Topological State 1.

**Figure 5:**
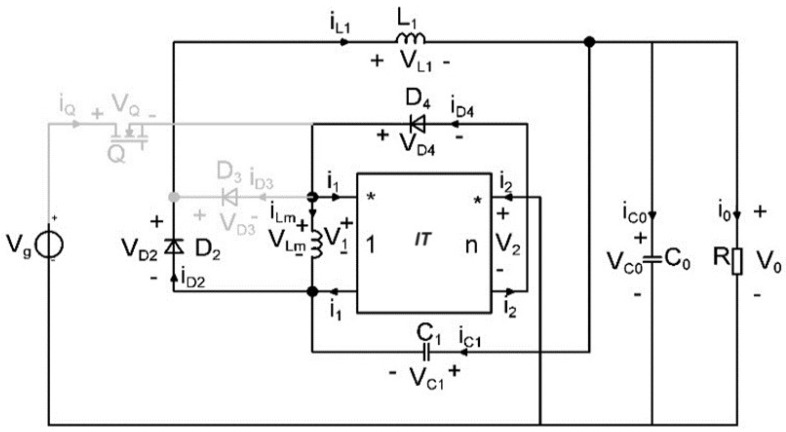
Equivalent model of the proposed buck−type converter with coupled inductors: Topological State 2.

In the first topological state, the transistor and diode *D*_3_ are conducting, while diodes *D*_2_ and *D*_4_ are blocked, while in the second topological state, with the transistor off, *D*_2_ and *D*_4_ are turned on, and diode *D*_3_ is off. The circuits corresponding to these two topological states are depicted in Figure 4 and Figure 5, respectively. Table 1 presents a resume of the switching states of the semiconductors.

Because of inductor coupling, an equivalent model that involves an ideal transformer denoted as *IT*, with the magnetizing inductor *L_M_,* is used. The dotted ports of the ideal transformer define the directions for the associated voltages and currents. The converter model with these substitutions is shown in Figure 3. It is important to highlight that the equation for the ideal transformer can be formulated as follows:

(1) $$v_{2}=n\cdot v_{1}$$

(2) $$i_{1}+n\cdot i_{2}=0$$

The voltage second balance principle is invoked for the purpose of determining both the DC capacitor voltages and the static conversion ratio, all within the structure of the small ripples assumption relating to the state variables.

The values of *V_C_*_1_, *V_C_*_0,_ and static conversion ratio, *M*, are obtained as [39]:

(3) $$V_{C1}=V_{g}\cdot \frac{D\cdot (1-D)}{1+n\cdot D}\;V_{C0}=V_{g}\cdot \frac{D\cdot (n+2-D)}{1+n\cdot D}\;M=\frac{V_{o}}{V_{g}}=\frac{D\cdot (n+2-D)}{1+n\cdot D}$$

**Figure 6:**
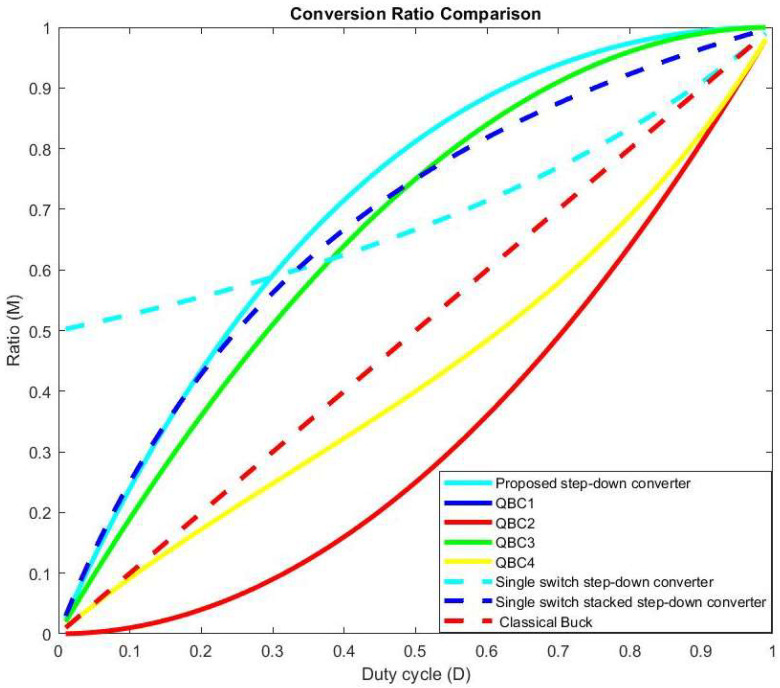
Conversion ratio comparison of the proposed step-down converter with other types of buck converters.

Upon careful consideration of Equation (3), it is obvious that the converter operates in a step-down mode, meaning it reduces the input voltage because *M* < 1. In order to provide a complete understanding, Figure 6 has been included, depicting a detailed comparison of the static conversion ratio between the proposed converter and the various types of step-down converters. This representation offers a clear understanding of the performance characteristics of the converters under study.

In order to obtain the inductor DC currents flowing through the inductors, equations representing charge balance are formulated, and the final result is [39]:

(4) $$I_{L1}=\frac{{D\cdot V}_{g}\cdot (n+2-D)}{R\cdot (1+n\cdot D)}$$

(5) $$I_{LM}=\frac{{D\cdot V}_{g}\cdot \left(1+n\right)\cdot (1-D)\cdot (n+2-D)}{R\cdot (1{+n\cdot D)}^{2}}$$

As observed, the proposed converter exhibits a characteristic similar to QBC [29] and stacked [33] converters. However, compared to the converter presented in [19], it shows a significantly better characteristic until the duty cycle reaches a value of 0.35.

To gain insight into how the proposed converter compares not only with other quadratic converters but also with other types of step-down converters, we have chosen to compare their key parameters. Table 2 reveals this comparison. As observed, the newly proposed converter has both advantages and disadvantages. The advantages are provided by the two degrees of freedom offered by the duty cycle and transformer ratio, as well as the relatively low stress on the transistor compared to other types of converters. The major disadvantage, taking the classic buck converter as a reference, pertains to the number of components, including the higher manufacturing cost and system complexity.

The stress on semiconductor elements, as well as component design, are also detailed in [39]. For inductor design, it was ordered that the current ripples be less than 25% of the DC value. For the inner capacitor design, it was imposed that the voltage ripples do not exceed 10% of the DC value, while for the output capacitor, this should not exceed 5%.

The step-down converter proposed is designed according to the following specifications:

Input voltage: *V_g_* = 30 V; 

Output voltage: *V_o_* = 18 V; 

Output power: *Po* = 10–15 W; 

Switching frequency: *fs* = 100 kHz; 

Transformer ratio: *n* = 0.66.

Using the MATLAB [41] program, the output resistor value is *R* = 33 Ω. The theoretical and simulated values of the magnetizing inductor, *L_M_*, which is equal to the value of *L*_2_, inductor *L*_3_, as well for the single inductor *L*_1_, inner capacitor *C*_1_, and output capacitor *C*_0_, are presented in the Table 3. The calculations performed in MATLAB provide the minimum values for the components; therefore, in the simulation, higher values were used.

**Figure 7:**
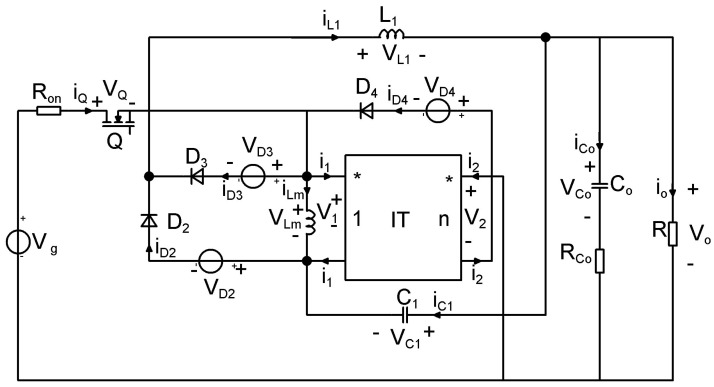
Equivalent model of the proposed buck−type topology, including losses.

**Figure 8:**
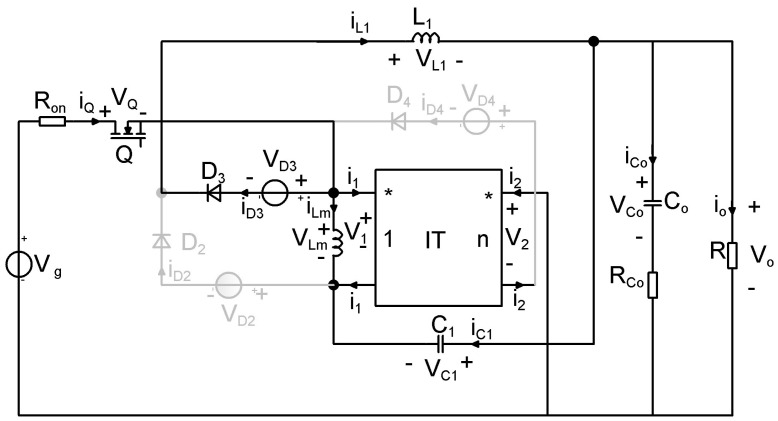
Equivalent lossy model of the proposed buck−type converter: Topological State 1.

**Figure 9:**
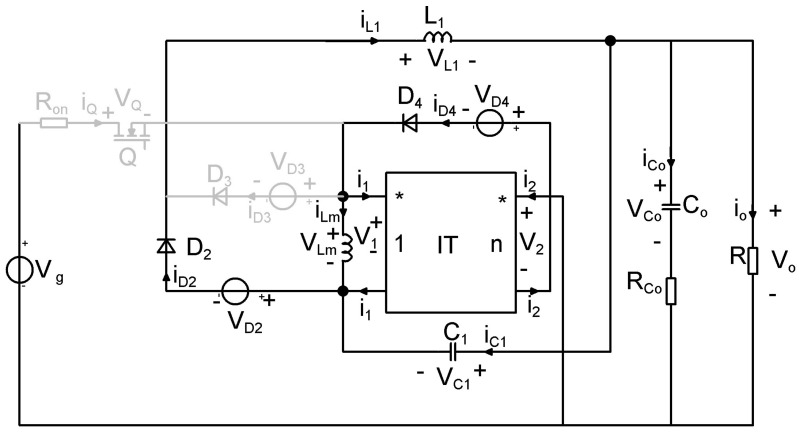
Equivalent lossy model of the proposed buck−type converter: Topological State 2.

To obtain the state matrices, the losses on the semiconductor elements as well as the losses on the output capacitor, are considered. The schematics containing lossy elements is represented in Figure 7 and the circuits corresponding to the two topological states in Figure 8 and Figure 9.

In order to determine the small-signal model of the proposed buck-type converter, first, the state–space equations for each topological state need to be determined. The state variables in the vector *x* are the inductor currents, $i_{L_{1}}$ and $i_{L_{M}}$ and the capacitor voltages, $v_{C_{1}}\;$ and $v_{C_{o}}$. The input vector *u* contains the supply voltage, $v_{g}$ and the three diodes forward voltage drops, $v_{D2}$, $v_{D3}$, $v_{D4}$ and the output vector *y* is the same as the state vector [36]. For easier tracking of calculations, the notation $R_{ech}$ was chosen for the grouping of parallel resistors at the converter’s output.

(6) $$R_{ech}=\frac{R\cdot R_{C0}}{R+R_{C0}}$$

The values for the duals of the state variables are:

(7) $$V_{LMon}=-R_{on}\cdot \left(i_{L1}\cdot i_{LM}\right)+V_{g}-R_{ech}\cdot \left(i_{L1}+i_{LM}\right)-\frac{R}{R+R_{C0}}\cdot V_{C0}+V_{C1}$$

(8) $$V_{L1on}=-V_{D1}-R_{on}\cdot \left(i_{L1}+i_{LM}\right)+V_{g}-R_{ech}\cdot \left(i_{L1}+i_{LM}\right)-\frac{R}{R+R_{C0}}\cdot V_{C0}$$

(9) $$I_{C1on}=-i_{LM}$$

(10) $$I_{C0on}=\frac{R}{R+R_{C0}}\cdot \left(i_{LM}+i_{L1}\right)-\frac{1}{R+R_{C0}}\cdot V_{c0}$$

From these equations, the derivatives of the state variables in terms of both state and input variables can be easily written, and from these scalar relationships, the matrices *A*_1_, *B*_1_, *E*_1,_ and *F*_1_ corresponding to the first topological state were determined:

$$A_{1}=\left[\begin{array}{cccc} -\frac{R_{on}+R_{ech}}{L_{M}} & \;-\frac{R_{on}+R_{ech}}{L_{M}} & \frac{1}{L_{M}} & -\frac{R}{L_{M}\cdot (R+R_{C0})}\\ -\frac{R_{on}+R_{ech}}{L_{1}} & -\frac{R_{on}+R_{ech}}{L_{1}} & 0 & -\frac{R}{L_{M}\cdot (R+R_{C0})}\\ -\frac{1}{C_{1}} & 0 & 0 & 0\\ \frac{R}{C_{0}\cdot (R+R_{C0})} & \frac{R}{C_{0}\cdot (R+R_{C0})} & 0 & -\frac{R}{C_{0}\cdot (R+R_{C0})} \end{array}\right]\;$$

(11) $$B_{1}=\left[\begin{array}{cccc} \frac{1}{L_{M}} & 0 & 0 & 0\\ \frac{1}{L_{1}} & -\frac{1}{L_{1}} & 0 & 0\\ 0 & 0 & 0 & 0\\ 0 & 0 & 0 & 0 \end{array}\right];E_{1}=\left[\begin{array}{cccc} 1 & 0 & 0 & 0\\ 0 & 1 & 0 & 0\\ 0 & 0 & 1 & 0\\ 0 & 0 & 0 & 1 \end{array}\right];F_{1}=\left[\begin{array}{cccc} 0 & 0 & 0 & 0\\ 0 & 0 & 0 & 0\\ 0 & 0 & 0 & 0\\ 0 & 0 & 0 & 0 \end{array}\right];$$

With a similar procedure, the matrices *A*_2_, *B*_2_, *E*_2,_ and *F*_2_ corresponding to the second topological state will be derived as:

(12) $$V_{LMoff}=-V_{D4}-R_{ech}\cdot \frac{i_{LM}}{1+n}-\frac{R}{R+R_{C0}}\cdot V_{C0}+V_{C1}$$

(13) $$V_{L1on}=-V_{D2}-V_{C1}$$

(14) $$I_{C1off}=i_{L1}-\frac{i_{LM}}{1+n}$$

(15) $$I_{C0off}=\frac{R}{R+R_{C0}}\cdot \left(\frac{i_{LM}}{1+n}\right)-\frac{1}{R+R_{C0}}\cdot V_{C0}$$

$$A_{2}=\left[\begin{array}{cccc} -\frac{R_{ech}}{{{\left(1+n\right)}^{2}\cdot L}_{M}} & 0 & \frac{1}{{(1+n)\cdot L}_{M}} & -\frac{R}{L_{M}\cdot (R+R_{C0})\cdot (1+n)}\\ 0 & 0 & \frac{1}{L_{1}} & 0\\ -\frac{1}{{(1+n)\cdot C}_{1}} & \frac{1}{C_{1}} & 0 & 0\\ \frac{R}{C_{0}\cdot (R+R_{C0})} & \frac{R}{C_{0}\cdot (R+R_{C0})} & 0 & -\frac{R}{C_{0}\cdot (R+R_{C0})} \end{array}\right]$$

(16) $$B_{2}=\left[\begin{array}{c} \;0\;\;\;\;\;\;\;\;\begin{array}{cc} \;\;0 & \;\;\;\;\;\;\;\;\begin{array}{cc} 0\;\; & -\frac{1}{L_{M}} \end{array} \end{array}\\ \begin{array}{c} \frac{1}{L_{1}}\;\;\;\;\;\;\;\;\begin{array}{ccc} 0 & \;\;\;\;-\frac{1}{L_{1}} & \;\;\;\;\;\;0 \end{array}\\ 0\;\;\;\;\;\;\;\;\begin{array}{ccc} 0 & \;\;\;\;\;\;\;\;\;0 & \;\;\;\;\;\;\;0 \end{array}\\ 0\;\;\;\;\;\;\;\;\begin{array}{ccc} 0 & \;\;\;\;\;\;\;\;\;0 & \;\;\;\;\;\;\;0 \end{array} \end{array} \end{array}\right];E_{2}=\left[\begin{array}{c} 1\;\;\;\;\;\;\;\begin{array}{cc} \;\;0 & \;\;\;\;\;\begin{array}{cc} 0\;\; & \;\;\;0 \end{array} \end{array}\\ \begin{array}{c} \;0\;\;\;\;\;\;\;\begin{array}{cc} \;\;1 & \;\;\;\;\begin{array}{cc} 0\;\; & \;\;\;0 \end{array} \end{array}\\ \;0\;\;\;\;\;\;\;\begin{array}{cc} \;\;0 & \;\;\;\;\begin{array}{cc} 1\;\; & \;\;\;0 \end{array} \end{array}\\ \;0\;\;\;\;\;\;\;\begin{array}{cc} \;\;0 & \;\;\;\;\;\begin{array}{cc} 0\;\; & \;\;1 \end{array} \end{array} \end{array} \end{array}\right];F_{2}=\left[\begin{array}{c} \;0\;\;\;\;\begin{array}{cc} \;\;0 & \;\;\begin{array}{cc} 0 & \;0 \end{array} \end{array}\\ \begin{array}{c} \;0\;\;\;\;\begin{array}{cc} \;\;0 & \;\;\begin{array}{cc} 0 & \;0 \end{array} \end{array}\\ \;\;0\;\;\;\;\begin{array}{cc} \;\;0 & \;\;\begin{array}{cc} 0 & \;0 \end{array} \end{array}\\ \;\;0\;\;\;\;\begin{array}{cc} \;\;0 & \;\;\begin{array}{cc} 0 & \;0 \end{array} \end{array} \end{array} \end{array}\right];$$

After averaging and linearization, the control-to-output function results as follows [42]:

(17) $$G_{c}\left(s\right)=E_{D}\cdot (s1-A_{D}{)}^{-1}\xi_{D}+\zeta_{D}$$

where

(18) $$A_{D}=D\cdot A_{1}+(1-D)\cdot A_{2}$$

(19) $$X=-A_{D}^{-1}B_{D}U$$

(20) $$\xi_{D}={(A}_{1}-A_{2})\cdot X+(B_{1}-B_{2})\cdot U$$

(21) $$\zeta_{D}={(E}_{1}-E_{2})\cdot X+(F_{1}-F_{2})\cdot U$$

Utilizing the above equations, the numerical control-to-output transfer function of the proposed converter is determined:

(22) $$G_{c}\left(s\right)=\frac{\;2.173e258\cdot \;s^{3}+1.718e263\cdot \;s^{2}+5.747e266\cdot \;s+5.165e271}{2.926e253\cdot \;s^{4}+9.228e256\cdot \;s^{3}+1.296e262\cdot \;s^{2}+2.733e265\cdot \;s+1.237e270}$$

Given that the control-to-output transfer function is of the fourth order, it involves a higher level of complexity in the controller design. This challenge can be addressed by simplifying the fourth-order control-to-output transfer function using a second-order approximation. Then, the controller can be designed based on this lower-order transfer function. It is important to mention that the original transfer function must be accurately approximated only within half of the switching frequency, as this falls within the valid domain of the average model. Using the tfest (estimate transfer function using frequency domain data, specifying the number of poles and the number of zeros for approximating the transfer function) command in MATLAB [41], the approximated control-to-output function with two poles and two zeros can be written as:

(23) $$G_{c}\left(s\right)=\frac{-0.3247\;s^{2}+8.49e04\cdot \;s+5.707e09}{s^{2}+3063\cdot \;s+1.372e08}$$

**Figure 10:**
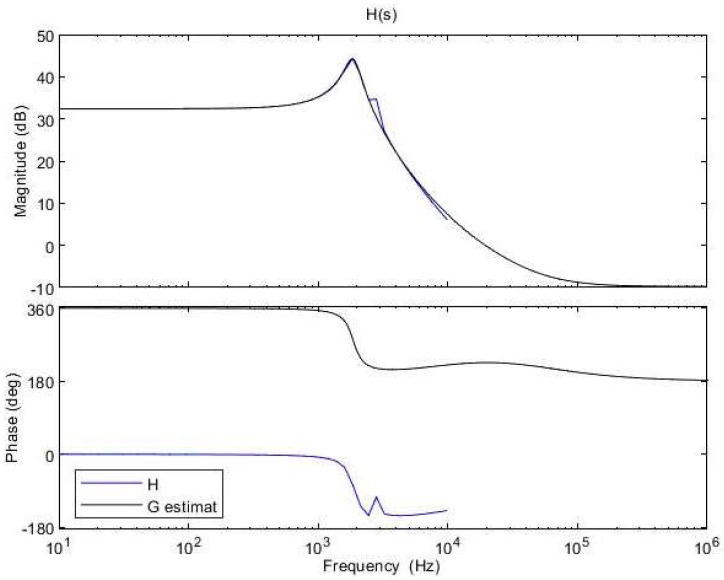
Initial control−to−output transfer function (blue) and rounded control−to−output transfer function (black).

In Figure 10, the original and the approximated transfer functions are illustrated, and the estimation data fit is 86.12%.

The chosen error amplifier for the controller’s design is of type III. To optimize performance, the compensation circuit must be ruinously modeled. This involves configuring it not only to provide a high DC gain but also to incorporate a phase “boost.” This approach is essential for obtaining a phase margin of sufficient magnitude. The transfer function of the ideal type III error amplifier is [42]:

(24) $$H_{AE}\left(s\right)=\frac{1}{\frac{s}{\omega_{UGF}}}\cdot \frac{\left(1+\frac{s}{\omega_{z1}})\cdot (1+\frac{s}{\omega_{z2}}\right)}{\left(1+\frac{s}{\omega_{p1}})\cdot (1+\frac{s}{\omega_{p2}}\right)}$$

By applying the pole-zero placement method as outlined in reference [42], the specific parameters of the error amplifier are determined. A crossover frequency of 9 kHz is chosen. For this purpose, a MATLAB 2021 script provided in the Appendix was developed. The results are:

(25) $$\omega_{ugf}=5.4379e+03\;rad/s$$

(26) $$\omega_{p1}=\omega_{ZESR}=55455rad/s$$

(27) $$\omega_{p2}=3.1416e+05\;rad/s$$

(28) $$\omega_{z1}=\omega_{z2}=\omega_{0}=11713\;rad/s$$

**Figure 11:**
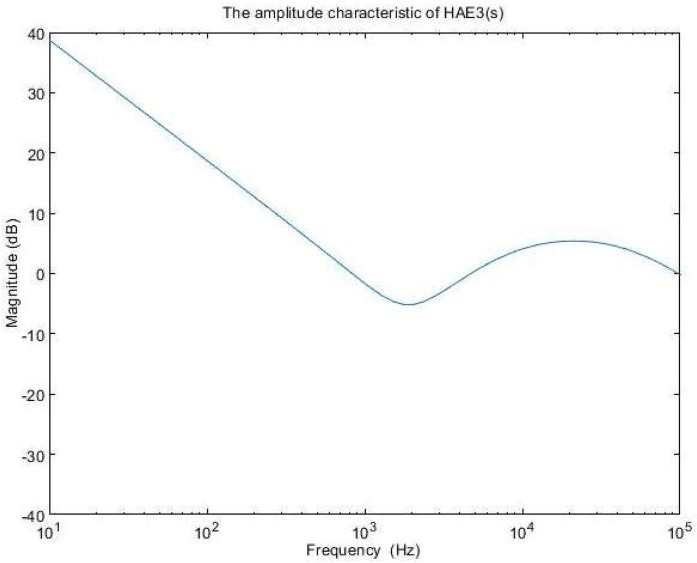
The amplitude characteristic of H_AE3_(s).

**Figure 12:**
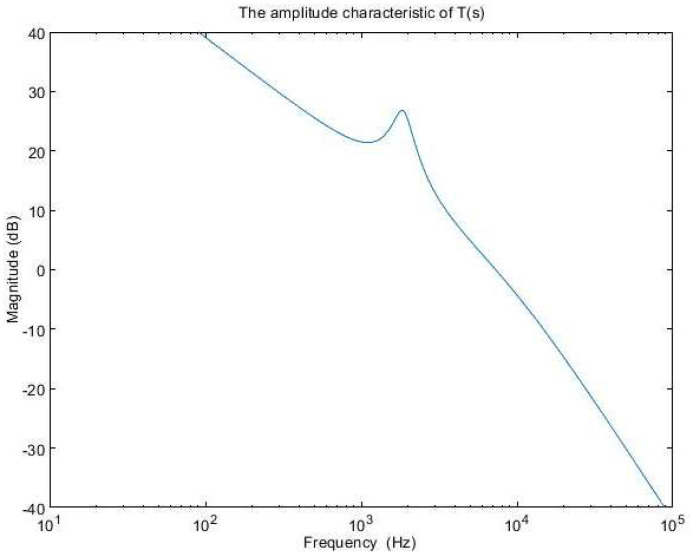
The amplitude characteristic of T(s).

**Figure 13:**
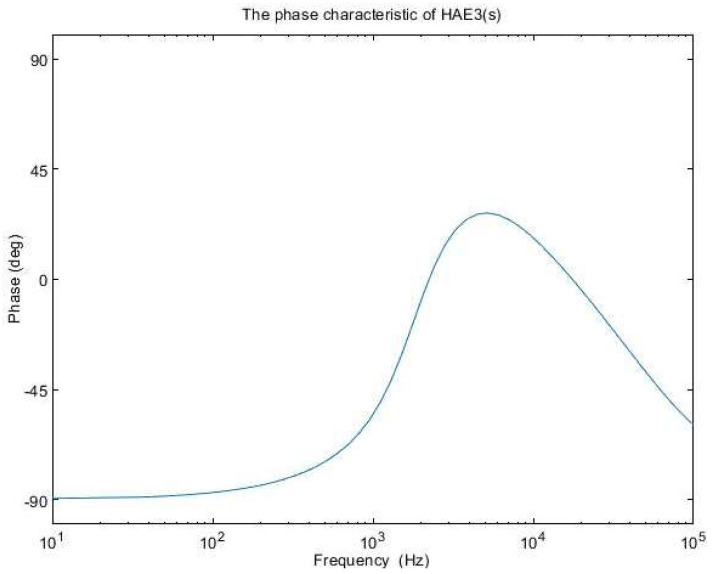
The phase characteristic of H_AE3_(s).

**Figure 14:**
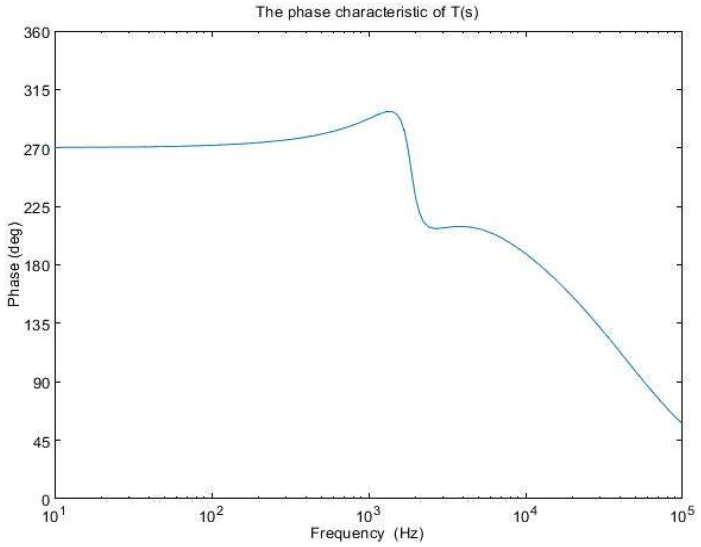
The phase characteristic of T(s).

The amplitude characteristics of the error amplifier with these values is presented in Figure 11, while the open-loop transfer function amplitude characteristic is depicted in Figure 12. The phase characteristic of the error amplifier is sketched in Figure 13 and the phase characteristic of the open-loop transfer function can be examined in Figure 14. Examining Figure 12, it can be remarked that the amplitude characteristic is monotonically decreasing with a slope of −20 db/decade, except for a peak given by the high-quality factor in the denominator of (23). The real crossover frequency is 7.3 kHz, and the phase margin results in 20 degrees. 

The theoretical assumptions will be validated through simulations in CASPOC [43]. The PWM signal controlling the transistor gate is characterized by switching frequency f_S_ = 100 kHz. All components, including transistors and diodes, are considered with losses. 

**Figure 15:**
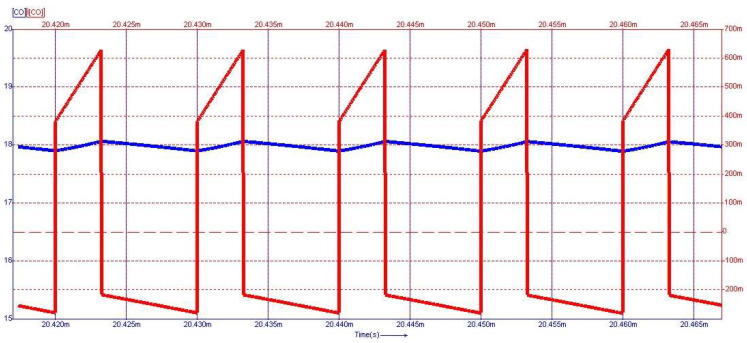
Voltage (blue) and current (red) waveforms for the output capacitor *C*_0_. Voltage regulation at 18 V is observed.

**Figure 16:**
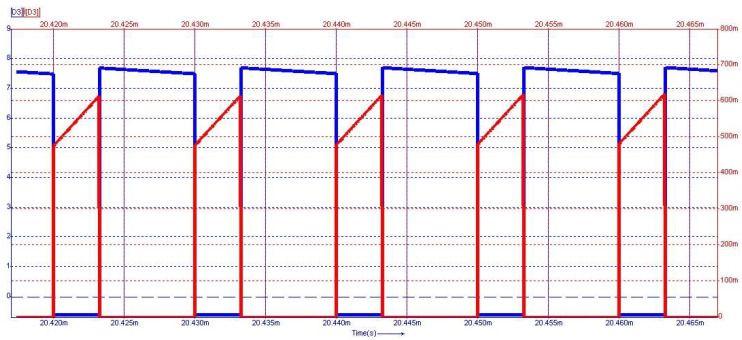
Voltage (blue) and current (red) waveforms for diode *D*_3_.

**Figure 17:**
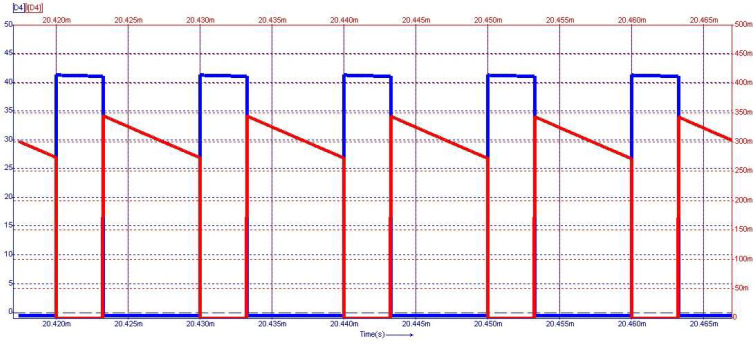
Voltage (blue) and current (red) waveforms for diode *D*_4_.

Figure 15 depicts the steady-state waveforms in closed-loop operation for the voltage across and the current through the capacitor *C*_0_. The output voltage was set to 18 V. The semiconductor voltages and currents for the diodes D_3_ and D_4_, which conduct in the first topological state, are shown in Figure 16, and the second topological state in Figure 17, respectively. 

**Figure 18:**
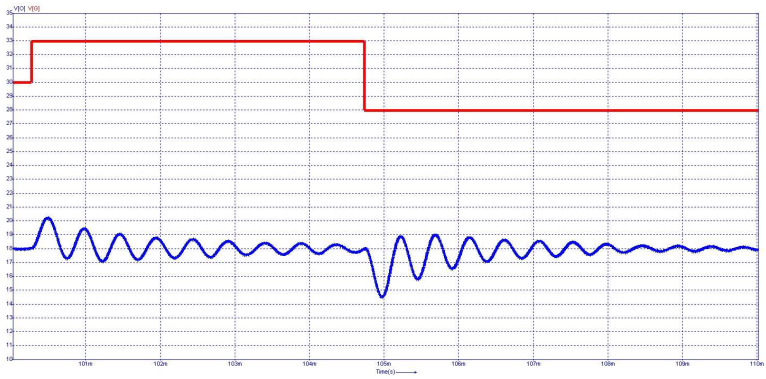
Dynamic behavior at step change in the input voltage: input voltage (red-*V_g_*), output voltage (blue-*V_o_*).

**Figure 19:**
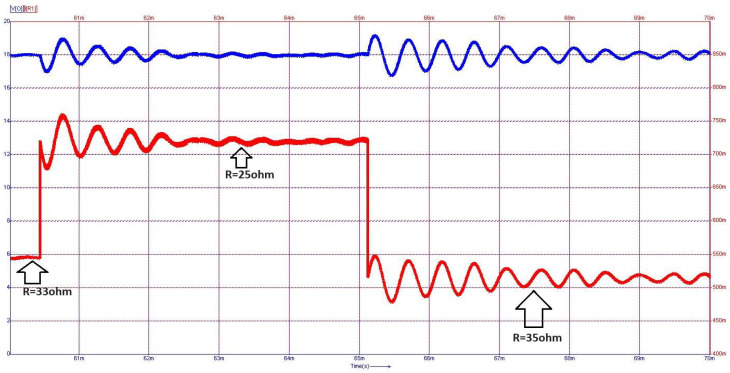
Dynamic behavior at step changes in the load resistance: output voltage (blue-*V_o_*), output current (red-*I_R_*_1_).

From Figure 15 to Figure 17, on the *Y*-axis-left side (blue color), the variation of voltage across the illustrated element is represented with the measurement unit in the international system [V]. Simultaneously, the *Y*-axis-right side (red color) depicts the waveform of the current through the component, represented in [mA]. Figure 18 shows the evolution of the output voltage at step changes in the input voltage that is modified from 30 V to 33 V and then to 28 V. It can be observed how, after the transient, the output voltage is regulated at the 18 V value described in the above requirement. In Figure 19, the converter behavior to step changes in the load resistance is presented. The initial load resistance is *R* = 33 Ω, then it is suddenly decreased to *R* = 25 Ω, and after some time, it is again increased to R = 35 Ω. Good regulation is observed in the output voltage revealed by the load current quasi-rectangular aspect. Each step change is accompanied by some ringing caused by the low phase margin value. A better transient response can be achieved by decreasing the crossover frequency at the expense of a longer response time. 

## 3. Results

The paper focuses on closed-loop operations and controller design for a fourth-order converter. As controller design for higher-order systems is cumbersome, after deriving the control-to-output transfer function based on a matrix state–space model, the authors approximated it by a second-degree transfer function. This approximation is accurate in the low-frequency domain, up to half the switching frequency. Then, for the second-order transfer function, a type III error amplifier is designed using the traditional pole-zero placement method. Upon analyzing the output results from the simulation, it becomes obvious that the transient behavior following a step change, even if it is in the input voltage or the output load, is notably restrained. This characteristic contributes to the overall stability of the system, although the fact that the approximation of the transfer function amounted to only 86.12%, the time response is short and validates the theoretical analysis.

## 4. Discussion

The method proposed by the authors can be extended to any converter of order three or four. Depending on converter parameters, the approximation by a second-order transfer function can have a better or a poor accuracy, but generally, good accuracy is provided by the tfest function in MATLAB. Dynamic behavior can be adjusted by modifying the crossover frequency and the phase margin. 

## 5. Conclusions

The main focus of the paper revolves around the intricate aspects of closed-loop operations and the design of controllers specifically tailored for a fourth-order converter. Given the challenges associated with developing controllers for systems of higher order, the authors begin with the derivation of the control-to-output transfer function established in a matrix state–space model, obtained for an approximation using a second-degree transfer function. This approximation demonstrates remarkable accuracy within the low-frequency domain, extending up to half the switching frequency.

Following this approximation, the authors proceeded to design a type III error amplifier for the second-order transfer function, applying the conventional pole-zero placement method. 

The conclusion using this approach is that the approximation method of the transfer function can indeed be truly useful for high-order converters. Even though the approximation percentage does not reach 100%, the result is a stable system that provides a fast response to triggers in the input voltage or changes in the output load.

## Figures and Tables

**Table 1:** Corresponding states of the converter.

Semiconductor Devices	State 1	State 2
Q	On	Off
D_2_	Off	On
D_3_	On	Off
D_4_	Off	On

**Table 2:** Comparison between several step-down type converters.

Parameter	Proposed	QBC1 [28]	QBC2 [27]	QBC3 [29]	QBC4 [30]	Single Switch [19]	Stacked [33]	Classical [13]
No. of transistor	1	1	1	1	1	1	1	1
No. of diodes	3	3	3	3	3	2	3	1
Total no. of components	9	8	8	8	8	8	12	4
System order	4	4	4	4	4	5	8	2
Static Conversion Ratio (M)	$\frac{D\cdot (n+2-D)}{1+n\cdot D}$	$D^{2}$	$D^{2}$	D·(2−D)	$\frac{D}{1+D-D^{2}}$	$\frac{1}{\left(2-D\right)}$	$\frac{n\cdot D}{\left(1+2\cdot D\right)}$	D
Transistor voltage stress	$\frac{V_{g}}{1+n\cdot D}$	$\left(1-\sqrt{M}+2\cdot M\right)\cdot V_{g}$	$V_{g}$	$V_{g}$	$\frac{1+D}{1+D-D^{2}}\cdot V_{g}$	$M\cdot V_{g}$	$\frac{1+D}{\left(1+2\cdot D\right)}\cdot V_{g}$	$V_{g}$
Transistor DC current stress	$M\cdot \frac{D\cdot n+2-D)}{\left(1+n\cdot D\right)}\cdot \frac{V_{g}}{R}$	$M\cdot \frac{V_{g}}{R}$	$M\cdot \frac{V_{g}}{R}$	$M^{2}\cdot \frac{V_{g}}{R}$	$M\cdot \frac{D}{\left(1+D-D^{2}\right)}\cdot \frac{V_{g}}{R}$	$2\cdot (2M-1)\cdot \frac{V_{g}}{R}$	$M\cdot \frac{3}{\left(1+2\cdot D\right)}\cdot \frac{V_{g}}{R}$	$M\cdot \frac{V_{g}}{R}$
Maximum diode voltage stress	$\frac{\left(n+1\right)}{1+n\cdot D}\cdot V_{g}$	$V_{g}$	$\surd M\cdot V_{g}$	$V_{g}$	$\frac{1+D}{1+D-D^{2}}\cdot V_{g}$	$M\cdot V_{g}$	$\frac{2\cdot D}{\left(1+2\cdot D\right)}\cdot V_{g}$	$V_{g}$
Maximum diode DC current stress	$M\cdot {\left(1-D\right)}^{2}\cdot \frac{V_{g}}{R}$	$M\cdot \frac{V_{g}}{R}$	$M\cdot \frac{V_{g}}{R}$	$M\cdot \frac{V_{g}}{R}$	$M\cdot \frac{\left(1-D\right)}{\left(1+D-D^{2}\right)}\cdot \frac{V_{g}}{R}$	$(1-M)\cdot \frac{V_{g}}{R}$	$M\cdot \frac{V_{g}}{R}$	$(1-M)\cdot \frac{V_{g}}{R}$

**Table 3:** Theoretical and simulated values of reactive elements.

Component	Theoretical Value	Simulated Value
Coupled inductor *L*_2_	398.84 µH	463 µH
Coupled inductor *L*_3_	173.73 µH	207 µH
Single inductor *L*_1_	264.83 µH	266 µH
Inner capacitor *C*_1_	3.07 µF	10 µF
Output capacitor *C*_0_	1.81 µF	10 µF

## Data Availability

Data are contained within the article.
